# Tissue Engineering the Annulus Fibrosus Using 3D Rings of Electrospun PCL:PLLA Angle-Ply Nanofiber Sheets

**DOI:** 10.3389/fbioe.2019.00437

**Published:** 2020-01-14

**Authors:** Alyah H. Shamsah, Sarah H. Cartmell, Stephen M. Richardson, Lucy A. Bosworth

**Affiliations:** ^1^Department of Materials, Faculty of Science and Engineering, The University of Manchester, Manchester, United Kingdom; ^2^Division of Cell Matrix Biology and Regenerative Medicine, Faculty of Biology, Medicine and Health, Manchester Academic Health Sciences Centre, School of Biological Sciences, University of Manchester, Manchester, United Kingdom; ^3^Department of Eye and Vision Science, Faculty of Health and Life Sciences, Institute of Ageing and Chronic Disease, University of Liverpool, Liverpool, United Kingdom

**Keywords:** electrospinning, annulus fibrosus, polycaprolactone, poly(L-lactic) acid, polymer blend, cell-sheet-rolling-system

## Abstract

Treatments to alleviate chronic lower back pain, caused by intervertebral disc herniation as a consequence of degenerate annulus fibrosus (AF) tissue, fail to provide long-term relief and do not restore tissue structure or function. The future of AF tissue engineering relies on the production of its complex structure assisted by the many cells that are resident in the tissue. As such, this study aims to mimic the architecture and mechanical environment of outer AF tissue using electrospun fiber scaffolds made from a synthetic biopolymer blend of poly(ε-caprolactone) (PCL) and poly(L-lactic) acid (PLLA). Initially, an aligned bilayer PCL:PLLA scaffold was manually assembled at ±30° fibers direction to resemble the native AF lamellar layers; and bovine AF cells were used to investigate the effect of construct architecture on cell alignment and orientation. Bilayer scaffolds supported cell adhesion and influenced their orientation. Furthermore, significant improvements in tensile stiffness and strength were achieved, which were within the reported range for human AF tissue. Electrospun bilayer scaffolds are, however, essentially two-dimensional and fabrication of a complete three-dimensional (3D) circular construct to better replicate the AF's anatomical structure is yet to be achieved. For the first time, a custom-built Cell Sheet Rolling System (CSRS) was utilized to create a 3D circular lamellae construct that mimics the complex AF tissue and which overcomes this translational limitation. The CSRS equipment is a quick, automated process that allows the creation of multilayered, tube-like structures (with or without cells), which is ideal for mimicking human cervical AF tissue in term of tissue architecture and geometry. Tube-like structures (6 layers) were successfully created by rolling ±30° bilayer PCL:PLLA scaffolds seeded with bovine AF cells and subsequently cultured for 3 weeks. Cells remained viable, purposefully oriented with evidence of collagen type I deposition, which is the main structural component of AF tissue. This is the first study focused on applying CSRS technology for the fabrication of a more clinically-relevant, 3D tissue engineered scaffold for AF tissue regeneration.

## Introduction

**Figure 1:**
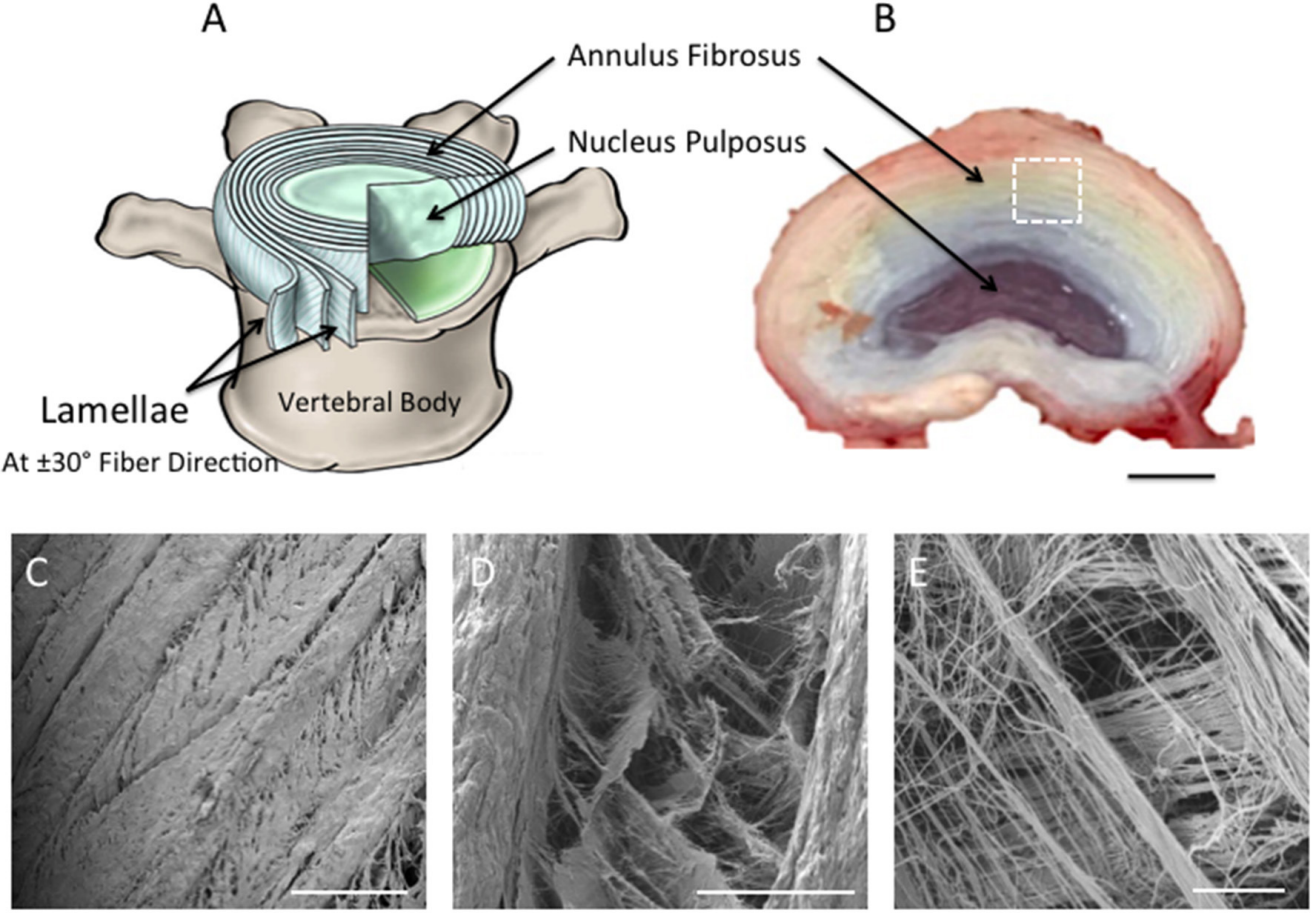
**(A)** Schematic diagram representing intervertebral disc in **(B)**. Note the nucleus pulposus (NP) at the center of the disc; the concentric rings (lamellae) of the AF are arranged in alternating fibers that are angled ~±30° from each other [**(A)** Adapted from Kandel et al. (2014)]. **(B)** Photograph taken of the superior view of dissected porcine lumbar intervertebral discs (aged 6-month) (Scale bar = 1 cm). **(C–E)** Scanning Electron Microscopy images demonstrating the superior view of the annular fibrosus lamellar layers. Images highlight the angle-ply fiber network between adjacent layers. **(C–E)** are magnified regions of interest observed within **(A)**, which are represented by the white dashed square (Scale bars: **C** = 500 μm at x65 magnification; **D** = 100 μm at x500 magnification; **E** = 10 μm at x2,700 magnification).

Treatments to alleviate chronic lower back pain, caused by intervertebral disc (IVD) degeneration and herniation, fail to restore IVD structure or function. Tissue engineering is a promising approach for the treatment of degenerative IVD tissues (van Uden et al., 2017). However, their complex architectural and mechanical properties make synthesis of a biomimetic, artificial substrate challenging. The disc has heterogeneous biphasic structure that consists of fibrous concentric lamellar sheets, the annulus fibrosus (AF), surrounding a gel-like material, the nucleus pulposus (NP) (Hickey and Hukins, 1980; Raj, 2008). In the AF region, each lamellar sheet is angularly oriented at 30° and alternatively to each other, while the collagen fibers within every lamella are aligned parallel to each other (Figure 1A). This cross-aligned fibrous structure is critical for complex mechanical behavior that has non-linear anisotropic properties. Under tension, the cross-aligned fibers in native AF tissue possess a uniform stiffness in the outer region that is essential for providing stability to the spinal motion segment (Skaggs et al., 1994; Holzapfel et al., 2005). Building on our previous work (Shamsah et al., 2019), structural evaluation of porcine IVD tissue, in particular the outer AF region, provided a detailed understanding of the tissue's architecture (Figures 1B–E), which can be incorporated into the design and development of synthetic scaffolds.

In order to mimic the properties of native AF using biomaterials, such as polymers, it is essential the scaffold closely replicates native structural architecture, including physical properties (Long et al., 2016). Thus, the method of scaffold fabrication must be carefully selected to ensure that the scaffold structure possesses aligned fibers at an appropriate scale in order to stimulate the desired cell response and guide matrix formation (van Uden et al., 2017; Buckley et al., 2018; Chu et al., 2018). Electrospinning is the ideal technique for fabrication of highly aligned fibrous scaffolds capable of mimicking the structural architecture of AF extracellular matrix (ECM). In addition, whilst it is important to replicate the tissue's architecture, it is also ideal to match the mechanical properties of the scaffold to those of the natural tissue as closely as possible. For the outer AF, the mechanical properties ideally need to possess a tensile strength and stiffness of ~10 MPa (Skaggs et al., 1994) and 136 MPa (Skaggs et al., 1994), respectively.

Polycaprolactone (PCL) electrospun fibers have been previously studied for tissue engineering due to their biocompatibility (Schutgens et al., 2015), long-term biodegradability (Bosworth and Downes, 2010), and mechanical properties that show resemblances to native AF tissue (Chen et al., 2013). However, PCL possesses low Young's modulus, which could consequently result in functional failure of the AF. To overcome this limitation, our previous work (Shamsah et al., 2019) described a strategy to improve this limitation by blending PCL with poly L-lactic acid (PLLA), a synthetic biocompatible biopolymer characterized by high mechanical modulus (Chen et al., 2013). The data suggested that, in addition to demonstrating biocompatibility, a bilayer scaffold made from a 50:50 blend of PCL and PLLA with opposing fibers (i.e., angle-ply structure) possessed similar structural and mechanical properties to human AF. This current study investigates the response of bovine AF cells seeded on ±30° bilayer fiber scaffolds and scalability of the blended scaffold into a more clinically-relevant 3D structure through the incorporation of an automated Cell Sheet Rolling System (CSRS) developed by Othman et al. (2015) to create controlled 3D tubular scaffold architectures.

## Materials and Methods

### Electrospinning, Scaffold Preparation, and Fiber Orientation

Polycaprolactone (PCL; inherent viscosity = 1.3 dL/g; Purac Biomaterials, Amsterdam, The Netherlands), and poly-L-lactic acid (PLLA; inherent viscosity = 3.2 dL/g; Purac Biomaterials, Amsterdam, The Netherlands), were dissolved at room temperature in 1,1,1,3,3,3-hexafluoro-2-propanol (HFIP; Sigma-Aldrich, UK) to prepare 10 and 5 %w/v solutions, respectively. From these, a 50:50 PCL:PLLA blended solution was prepared. Before electrospinning, ~0.1 g of rhodamine powder (Sigma-Aldrich, UK) was directly added into the solution to stain the fibers with an inorganic red dye to assist fluorescent imaging. Aligned electrospun nanofibrous scaffolds were collected using a single needle targeted toward a rotating mandrel (width = 120 mm, diameter = 30 mm) using a custom-built electrospinner with the following parameters: continuous flow rate (Cole-Parmer, St. Neots, UK): 1 mL/h, tip-to-collector distance: 20 cm, applied DC voltage (Glassman High Voltage, Reading, UK): 20 kV, and mandrel speed: 600 rpm.

Samples for the *in vitro* study were cut from the collected fiber sheet into 22 × 5 mm^2^ rectangles with fibers' angled at 30° relative to the circumferential axis of the mandrel. Electrospun fiber scaffolds were individually mounted on stainless steel stubs with carbon tabs (Agar Scientific, UK) and coated with platinum (10 nm thickness). Fiber orientation from the main direction (*n* = 120) was determined from low magnification SEM images (x1.8 k) using ImageJ software (1.48v) as previously described by Shamsah et al. (2019) and Abrámoff et al. (2004).

Due to the delicate nature of nanofiber scaffolds, PCL:PLLA blend scaffolds were individually mounted within a custom-made, portable frame created from strengthened aluminum foil sheets (0.08 mm thickness; Simpac, Glasgow, UK), which enabled easy handling and transportation of the scaffold for subsequent *in vitro* testing. Being heat-resistant, frames were autoclaved for 1 h. Once cool, electrospun samples were positioned over the frame using sterile forceps and secured in position by folding over the two extension arms.

#### *In vitro* Cell Seeding and Culturing on Bilayer Fiber Scaffold

AF cells were isolated from fresh bovine tail discs (18–36 months old) obtained from a local abattoir. The discs were excised and the outer AF tissue macroscopically dissected. Serum-free media containing 0.5% pronase (Merck Chemicals Ltd, Nottingham, UK) was used to enzymatically digest the tissue fragments for 1 h. Tissues were then transferred to serum-free media containing 0.5% collagenase type II (Invitrogen, UK) and 0.1% hyaluronidase (Sigma, UK) for 2–3 h on an orbital shaker at 37°C. Tissue debris was removed by filtering the supernatant through a 40 μm filter. Cells were collected following centrifugation at 500 *G* for 5 min and the cell pellet subsequently plated out and expanded to passage 3 at 37°C and 5% CO_2_ in 75 cm^2^ sterile flasks with Dulbecco's Modified Eagle's Medium (DMEM) containing 4.5 g/L glucose, 5% sodium pyruvate 10% FBS, 1% antibiotic, and 50 g/mL ascorbic acid (Gibco, Massachusetts, USA). PCL:PLLA scaffolds held within sterilized portable frames were placed into 6-well plates (ThermoFisher, Waltham, USA). Scaffolds were disinfected in 70 %v/v ethanol in distilled water and pre-wetted in culture media for 12 h. This media was removed and 200 μL of AF cell suspension (1 × 10^5^ cells/sample) was evenly distributed over the surface of each scaffold. Samples were left undisturbed in the incubator (Jencons-PLS, Bedfordshire, UK) for 30 min to allow initial cell attachment and a further 2 ml of media added. Samples were cultured for 2 days, after which two single-layer scaffolds seeded with cells were manually brought into apposition with each other to create a cellular bilayer scaffold with nanofibers lying at ±30**°** and where cells on the bottom layer were in direct contact with the underlying surface of the top layer. Bilayers were incubated for 2 weeks, with media changes every second day.

#### *In vitro* Cell Orientation on Bilayer Fiber Scaffold

Cell orientation was assessed at 1, 7, and 14 days using SEM and confocal microscopy. For SEM (Hitachi S3000N VPSEM, Berkshire, UK), samples (*n* = 2) were washed in PBS (Sigma-Aldrich, UK) and fixed in 2.5 %v/v glutaraldehyde in PBS at 4°C for 2 h. As previously described (Shamsah et al., 2019), samples were dehydrated through increasing concentrations of ethanol in distilled water (50–100 %v/v), chemically dried in hexamethyldisilazane (Sigma-Aldrich, UK), mounted on carbon-tabbed stubs, and gold-sputter coated. In order to image the cells positioned in between the bilayer scaffold, several small cuts were made on the top layer using a scalpel, which allowed the upper surface to be carefully removed using forceps to reveal the cell layer below. This allowed representative images of the cells present on the bottom layer of the bilayer scaffold to be captured. For confocal microscopy (Leica SP8, Leica Biosystem UK Ltd, UK), samples with live AF cells were washed with PBS, stained in the dark with calcein solution (1 μl calcein solution to 2.5 ml PBS) (Fluorescence-based Molecular Probes, ThermoFisher, Waltham, USA), and incubated at 37°C for 20 min. Samples were subsequently washed with PBS (Sigma-Aldrich, UK) and placed on glass coverslips for imaging.

#### Tensile Testing of Cellular Bilayer Scaffold

Cellular bilayer scaffolds and acellular bilayers were fixed for 30 min in 10% neutral buffer formalin (Sigma-Aldrich, UK) for tensile testing (*n* = 8). Cellular bilayer samples were tested and analyzed for tensile properties as outlined previously (Shamsah et al., 2019). Briefly, samples were mounted onto paper windows with sticky tape. Paper windows were gripped within tensile grips of an Instron (Model 1122) (Instron, Buckinghamshire, UK), allowing a 20 mm gauge length and the paper sides subsequently cut. Uniaxial tension was applied using a 10 N load cell and 0.1% strain rate. Distilled water was periodically sprayed over each sample to prevent it drying out during testing.

Stress-strain curves were obtained for each sample and, using the cross-sectional area of each sample, the Young's modulus, ultimate tensile strength, and maximum percentage strain were calculated. The modulus was determined by comparing two stress-strain points in the linear region of the curve; ultimate tensile stress and maximum strain were taken from the highest points reached on the curve.

### Fabricating 3D AF Tissue Biomimics Using CSRS

**Figure 2:**
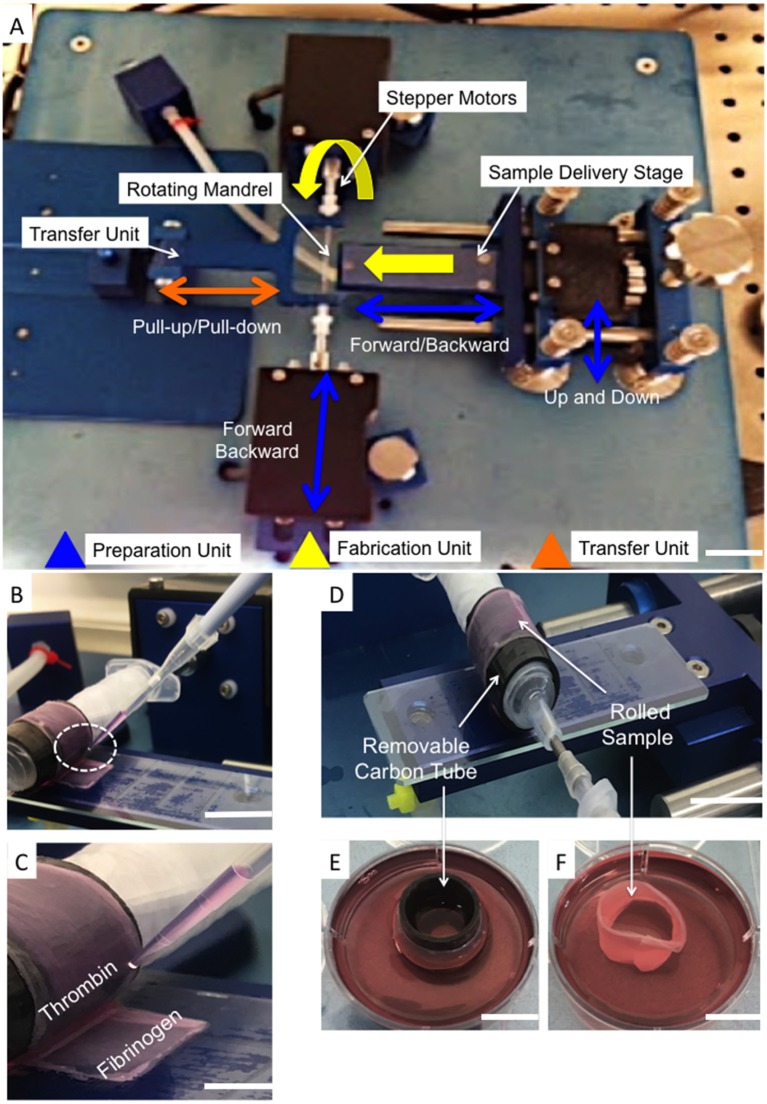
**(A)** Photograph of the automated Cell Sheet Rolling System (CSRS) developed by Othman et al. (2015). The main working units are highlighted: sample delivery stage; mandrel units (two stepper motors to rotate the mandrel), and tubular scaffold transfer units. **(B,C)** Photographs demonstrating the addition of fibrinogen-thrombin glue before completing the last rotation. **(D)** Photograph demonstrating the rolled sample after completing the last of 3 rotations onto the customized mandrel. **(E)** The carbon tube was securely transported to sterile well plates containing cell culture medium **(F)**. The ringed lamellar construct following careful removal of the carbon-tube (Scale bar **A,E,F** = 5 cm; **D,B** = 3 cm; and **C** = 1 cm).

Single layers of electrospun aligned fiber scaffolds were cut into 100 × 4 mm rectangles with fibers lying 30° to the longer length. Scaffolds were placed into pre-sterilized rectangular 4-well plates (ThermoFisher, Waltham, USA), disinfected and pretreated as previously described (section *in vitro* Cell Seeding and Culturing on Bilayer Fiber Scaffold), prior to cell seeding (2 × 10^5^ cells/sample). After 2 days, bilayer scaffolds were created as previously described (section *in vitro* Cell Seeding and Culturing on Bilayer Fiber Scaffold). After a further 2 days, cellular bilayer scaffolds were carefully removed from the culture media and individually placed onto sterile glass slides (ThermoFisher, Waltham, USA). An automated cell-sheet-rolling system (CSRS) was on loan from the medical engineering unit, University of Nottingham (Nottingham, UK) (Othman et al., 2015). The slide was placed onto the stage unit of the cell sheet rolling system (CSRS) and brought into contact with the mandrel that was housed within a removable carbon tube to give an outer diameter of 140 mm, which was purposefully selected to match the dimensions of the human outer AF tissue (Busscher et al., 2010; Weiler et al., 2012) (Figure 2A). Using the automation control unit, the stage advanced toward the mandrel at a speed that matched that of mandrel rotation, which thus allowed the smooth transfer and rolling of the scaffold around the mandrel. To minimize the risk of infection, the rolling process was performed within a Class II biological safety cabinet.

To avoid disassembly of the layers during culture, winding of the bilayer scaffold was temporarily paused to allow a thin layer (20 μm) of fibrin glue (Sigma-Aldrich, UK) to be applied to the inside of the final section to be rolled. Automation was subsequently restarted and the last rotation completed (Figures 2B,C). Once the rolling was complete (Figure 2D), the carbon tube was detached from the mandrel and the rolled sample carefully removed from this inner tubing. Rolled samples were immediately placed upright into 6-well plates containing 5 ml medium (Figures 2E,F) and cultured at 37°C, 5% CO_2_; media changes were performed every second day. After 3 and 21 days incubation the lamellar tubes were removed for evaluation.

#### *In vitro* Cell Response to 3D AF Tissue Biomimics

##### Engineered AF tissue biomimic measurements

Using a metric ruler, the height and diameter of the engineered AF (*n* = 5) were measured directly after rolling and after 3 and 21 days in culture. For each sample, the height was measured as the distance between the superior and inferior borders, which was taken at four different regions of the scaffold; and the diameter measured as the maximum distance between two lateral ends of each sample. The data were statistically analyzed using Graphpad Prism software (v8.1.1) and were presented as mean ± standard deviation.

*Live/dead assay*. Live/Dead assay kit (ThermoFisher, Waltham, USA) was used to examine cell viability and was performed as recommended by the manufacturer's protocol. Briefly, a working solution was prepared in the dark by adding 2.5 μl ethidium solution into 1 μl calcein solution, and both added to 2.5 ml PBS (Sigma-Aldrich, UK). The prepared solution was added to the samples (*n* = 3) and incubated at 37°C for 20 min. The samples were washed twice with PBS and directly transferred onto glass coverslips for 3D imaging using laser confocal microscopy (Leica TCS SP5, Leica Biosystem UK Ltd, UK). Projected z-stacked images of live and dead cells were obtained (3 representative images per sample) and the number of cells subsequently counted using ImageJ software (v1.48, LI-COR Technology, Nebraska, NE, USA). Cell viability then was evaluated using the mean value of cell count to estimate the percentage of live cells in a whole population.

*Alamar blue assay*. Alamar blue was used to measure the metabolic activity of AF cells at 3 and 21 days on the engineered construct (*n* = 3). Resazurin salt (Sigma-Aldrich, UK) was dissolved in PBS at a concentration of 0.125 mg/mL and filter-sterilized to create a stock Alamar blue solution. A 1:9 dilution of stock solution was added to fresh media in each well and incubated for 4 h. For each sample, 200 μL (in triplicate) was transferred to a 96-well plate and read by a fluorescence plate reader (Biotek, Swindon, UK) at 530 nm excitation and 590 nm emission. The number of metabolic cells directly correlates with the fluorescent intensity of the reduced resorufin and is expressed as mean fluorescence wavelength (nm) ± standard deviation.

*PicoGreen assay*. PicoGreen assay kit (Invitrogen, Massachusetts, USA) was used to detect changes in DNA quantity and was performed as per the manufacturer's protocol. In brief, samples (*n* = 3) were cut into small fragments, transferred to micro-tubes, centrifuged at 1,300 rpm with 1.5 ml lysis buffer (Invitrogen, Massachusetts, USA), and vortexed for 2 min to encourage break down of the cells' membrane. In 24-well plates (ThermoFisher, Waltham, USA), 2 μl of each sample added to 198 μl of 1X TE buffer (Tris: EDTA -Ethylene Diamine Tetra Acetic acid), which was previously prepared from 20X TE stock buffer as provided. A 1:200 dilution of PicoGreen reagent in 1X TE buffer was prepared and for each sample a volume of 100 μl was added to 100 μl sample volume (in triplicate) in a 96 black well plate (ThermoFisher, Waltham, USA). Samples were incubated in the dark at room temperature (5 min) and read using a fluorescent plate reader (Biotek, Swindon, UK) with 485 nm excitation and 535 nm emission. The content of DNA directly correlates with the fluorescent intensity and is expressed as mean fluorescence wavelength (nm) ± standard deviation.

#### Cell Distribution

The distribution of cells across the tubular lamellar construct was imaged using a nuclear stain. Samples were fixed (*n* = 2) with 10% neutral buffered formalin (Fisher Scientific, Waltham, USA) at room temperature for 30 min, washed twice with PBS, then 300 μl of Hoechst 33342 solution (20 mM; Life Technologies) was added to each sample and then incubated in the dark at room temperature for 5 min. Samples were subsequently washed several times in PBS (Sigma-Aldrich, UK) and mounted onto glass slides for imaging using laser confocal microscopy (Leica SP8, Leica Biosystem UK Ltd, UK).

#### Fibers and Cell Orientation

Fiber alignment across acellular tubular lamellar constructs was imaged using SEM (Hitachi S3000N VPSEM, Berkshire, UK) as described previously in section *in vitro* Cell Orientation on Bilayer Fiber Scaffold. Similarly, fiber alignment (Rhodamine stained) and cell orientation (Hoechst 33342 nuclear stain and molecular calcein stain for live cells) across cellular tubular lamellar constructs were imaged using laser confocal microscopy (Leica TCS SP5, Leica Biosystem UK Ltd, UK) as described previously in sections *in vitro* Cell Orientation on Bilayer Fiber Scaffold and Cell Distribution.

#### Detection of ECM

Specific detection of collagen type I was achieved by immunohistochemistry staining. Samples (*n* = 2) were fixed in 3 %v/v glutaraldehyde in PBS (Sigma-Aldrich, UK) for 30 min at room temperature, washed twice in PBS, then sectioned (5 μm) using a Leica C3050s cryostat. Slides were racked up in Sequenza (Thermo-Scientific) to avoid drying. They were then permeabilized with 0.1% Triton X-100 (Thermo-Scientific) in PBS for 1 h, and blocked with 1% bovine serum albumin (Sigma-Aldrich, UK) in PBS for 5 min at room temperature. Samples were washed twice with PBS before adding 2.5% Goat Serum (Invitrogen) in PBS for 10 min. Slides were washed with PBS (5 min x2) and stained with primary antibody (collagen I, ab90395, abcam) at 1:2,000 for 1 h, followed by repeated PBS washing and subsequent staining with the secondary antibody (AlexaFluor goat anti-mouse 546, 1:1,000) in distilled water (ThermoFisher, Waltham, USA) and incubated in Sequenza for 30 min. Slides were washed with PBS (5 min x2), before cleaning and covering with a coverslip and subsequently imaged under laser confocal microscopy (Leica TCS SP5, Leica Biosystem UK Ltd, UK).

### Statistical Analysis

The data were statistically analyzed using Graphpad Prism software (v8.1.1) and checked for normality. Normally distributed data were presented as mean ± standard deviation. Statistical analyses were performed using two-way ANOVA with multiple comparisons Tukey's tests, or unpaired *t*-test and Welch's correction *(p* < 0.05 was considered significant and has been defined with either a ^*^ or a down-caps line).

## Results

### The PCL:PLLA Bilayer Scaffold

#### *In vitro* Cell and Fiber Alignment on Bilayer Fiber Scaffold

**Figure 3:**
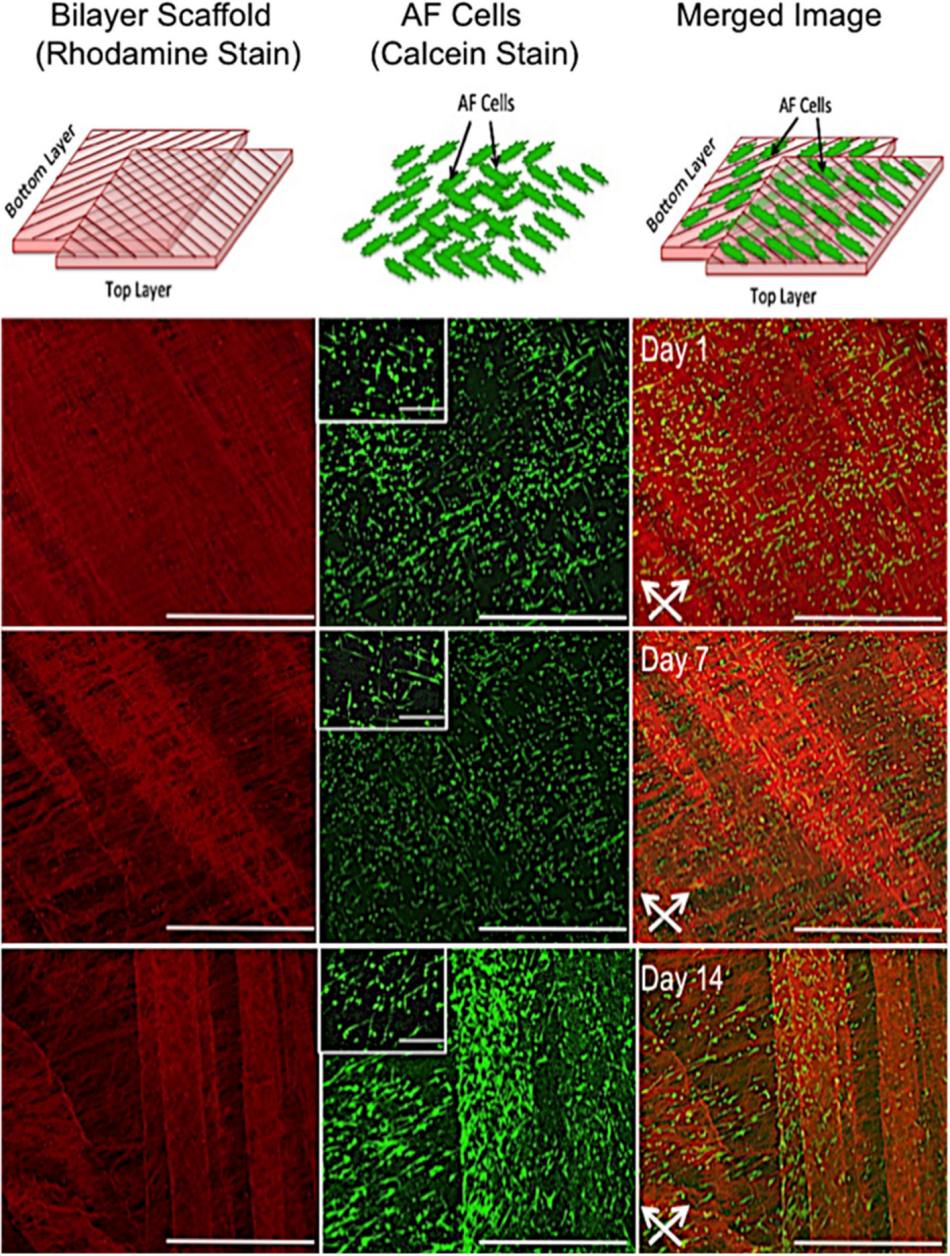
*In vitro* response of annulus fibrosus (AF) cells on PCL:PLLA electrospun bilayer scaffolds. Schematic diagrams representing the confocal microscopy image of Rhodamine fibers (red) with criss-cross direction; AF live cells (green) stained with Calcein; and merged image of both cells and fibers. Confocal microscopy images represent cell alignment at low and high magnifications within and on the bilayer scaffolds at day 1, 7, and 14 in culture [Scale bars: low magnification images (x60) = 750 μm; high magnification (inset) images (x500) = 100 μm]. White arrows indicate main direction of underlying fibers.

Confocal microscopy images representative of cell orientation within and on the bilayer scaffolds after 14 days in culture are shown in Figure 3. The crisscross arrangement of fibers (i.e., ±30°) was evident from the inclusion of rhodamine (red) during the electrospinning process. Calcein staining of live cells demonstrated their parallel alignment to the underlying fibers, which also presented an overall crisscross configuration. In addition, during the 14 days in culture the number of cell increased, which demonstrates cells were actively proliferating on the scaffold.

**Figure 4:**
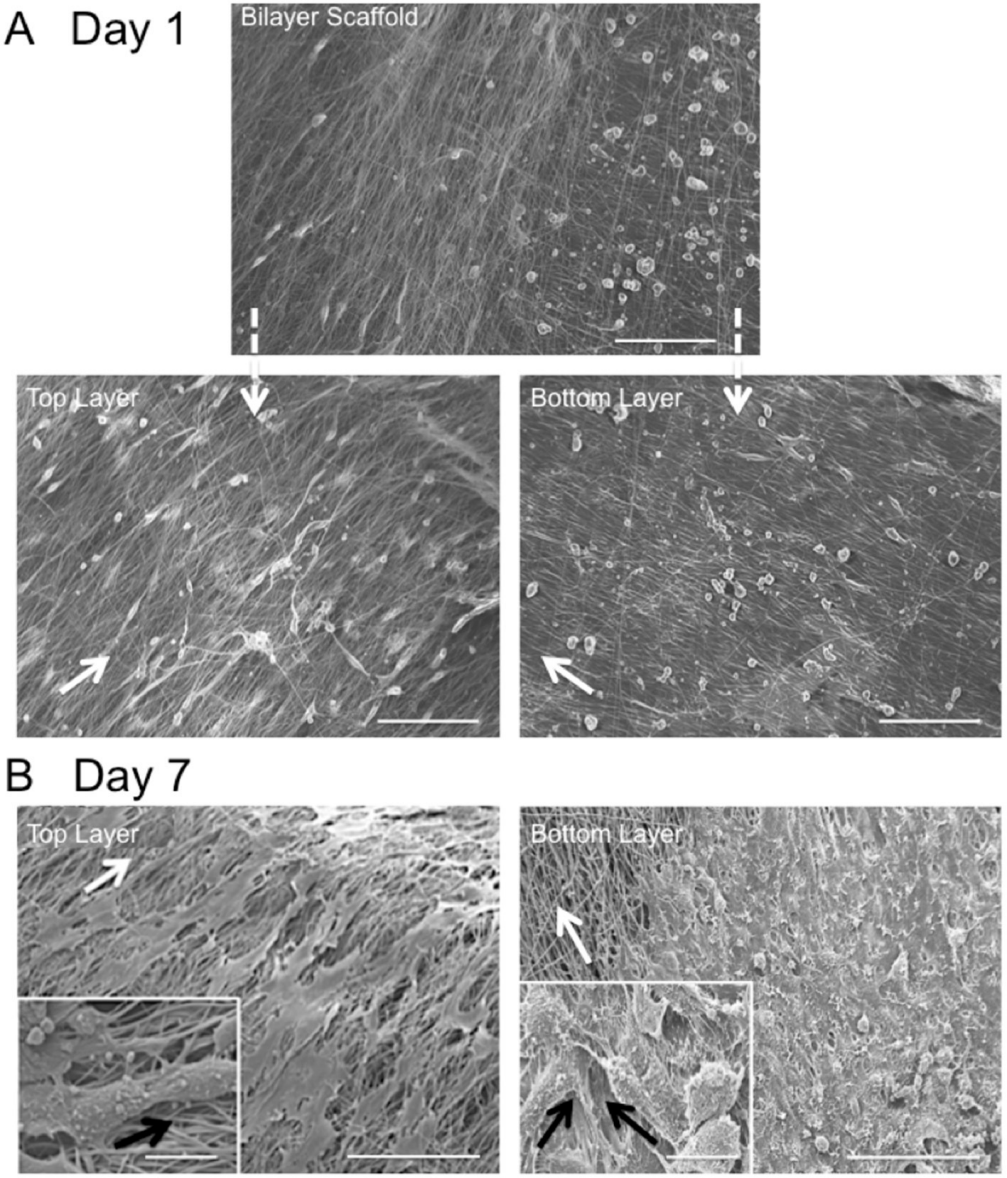
Scanning electron microscopy images of *in vitro* AF cell alignment on PCL:PLLA electrospun bilayer scaffolds after **(A)** 1 (magnification x270; Scale bar = 100 μm) and **(B)** 7 days in culture (main images represent low magnification (x370; Scale bar = 100 μm); inset images represent high magnification (x1,500) (Scale bar = 20 μm). White arrows indicate main fiber direction. Black arrows indicate cells' direction.

Fiber alignment relative to the main direction of the rotating mandrel was measured and a mean value of 33 ± 7° determined (data not shown). SEM images further demonstrated cell alignment on the surface of both scaffold layers after 7 days in culture (Figure 4A). In order to image the cells present on the bottom layer (i.e., within the middle of the bilayer scaffold), the top layer was carefully peeled off using tweezers. As evident from the high magnification images in Figure 4B, the morphology of AF cells on the top layer clearly appeared more elongated than those on the bottom layer, where cells demonstrated both rounded and aligned morphologies. It was also evident that some cells were aligned in opposing directions to others, which may have been due to their previous contact with the upper fiber layer, where fibers lay in a perpendicular direction.

#### Tensile Testing of Cellular Bilayer Scaffold

**Figure 5:**
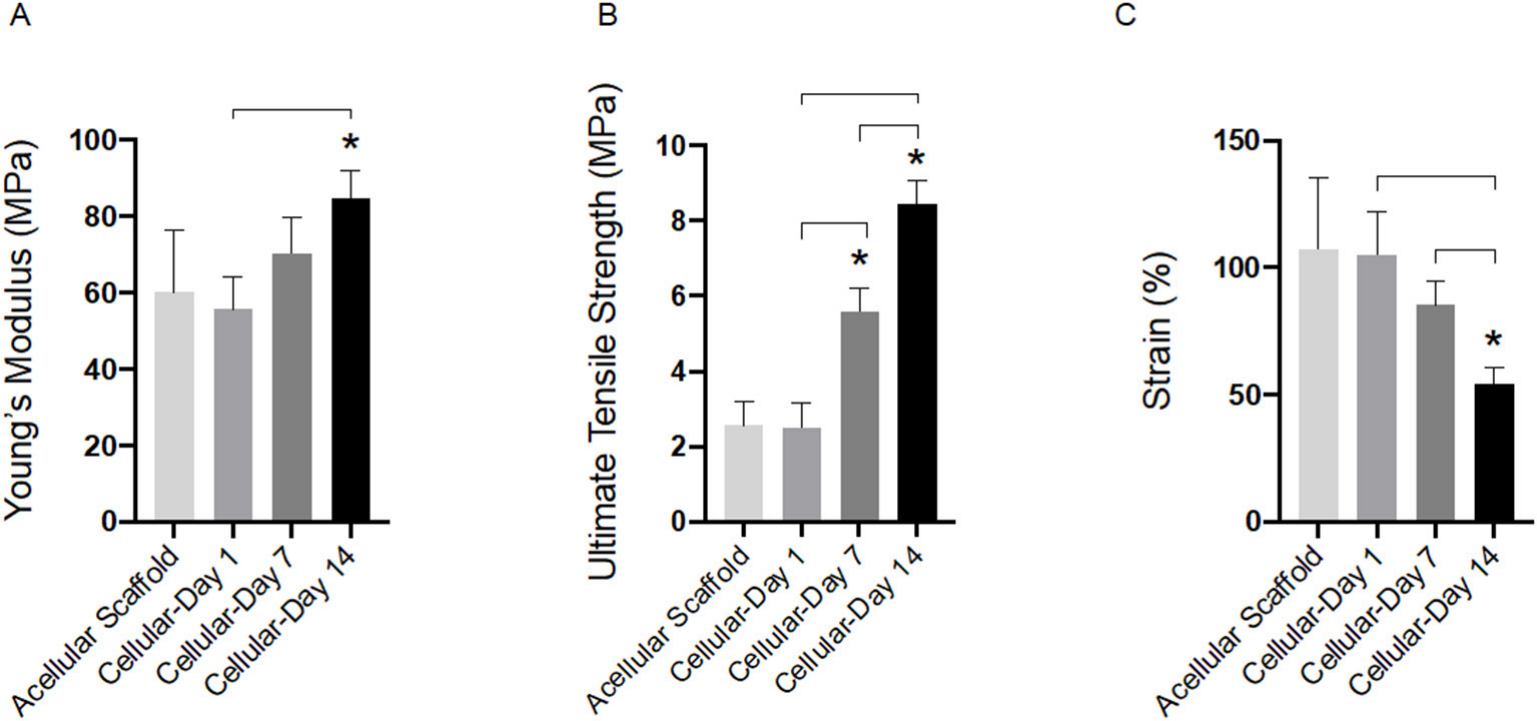
Tensile properties for acellular and cellular aligned electrospun bilayer scaffolds cultured for 1, 7, and 14 days with bovine AF cells; **(A)** Young's modulus, **(B)** Ultimate tensile strength, and **(C)** Percentage strain. Statistical comparison performed with two-way ANOVA and Tukey's post-test (*p* < 0.05; *n* = 8). Stars (*) represent statistical significance compared to acellular scaffold; down-caps line represents statistical significance between cellular scaffolds at different time points.

Tensile properties of cellular bilayer scaffolds were evaluated over 14 days in culture and compared to acellular scaffold controls. As shown in Figure 5A, there was no detected difference between the modulus of the acellular scaffold (60.0 ± 16.0 MPa) compared to cellular scaffolds after 1 and 7 days (55.6 ± 8.6 and 70.3 ± 9.2 MPa, respectively). However, presence of AF cells and suspected deposition of extracellular matrix resulted in the stiffness of the cellular bilayer scaffold to increase significantly after 14 days (84.7 ± 7.0 MPa) in culture compared to day 7 (70.3 ± 9.2 MPa) and by 41% compared to the acellular group (60.0 ± 16.0 MPa). There was no difference in ultimate tensile strength between acellular scaffold (2.5 ± 0.6 MPa) and cellular scaffold after day 1 (2.6 ± 0.7 MPa). However, a significant increase in strength was detected after 7 days (5.6 ± 0.7 MPa), followed by greatest strength at day 14, reaching 8.4 ± 0.7 MPa (Figure 5B). Bilayer scaffolds demonstrated a reduction in maximum strain over time, falling to 54% by 14 days, a difference of 65% compared to acellular controls (Figure 5C).

### The PCL:PLLA 3D Rings as AF Tissue Biomimics

#### Engineered Tissue Measurements

**Figure 6:**
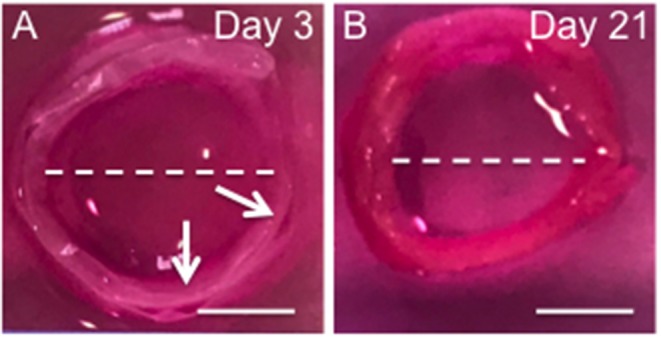
Photographs the 3D ringed lamellar construct after 3 **(A)** and 21 days culture **(B)**. Both samples feature a lamellar tubular structure, with slight delamination present between layers of day 3 sample. The white arrow indicates regions of delamination. The while dotted line indicates the maximum length used to measure internal diameter (Scale bars = 1 cm).

The Cell Sheet Rolling System (CSRS) was used create AF tissue biomimics by rolling 2D bilayer scaffolds into 3D ringed structures. Structural similarities between samples in terms of 3D circular shape were observed at all time points. The height and diameter of the fabricated tubes were measured immediately following rolling [time zero (0)] and at 3 and 21 days using a metric rule (Table 1 and Figure 6). The internal diameter following 3 days in culture was 14 ± 0.1 mm with a maximum height of 3.8 ± 0.1 mm. These dimensions had reduced by day 21, with the height decreasing by 18.4% and diameter 20.0% when compared to day 3. Slight delamination of the layers was observed at 3 days in culture; however, no delamination was present by day 21.

**Table 1:** Dimensions of tubular lamellar construct immediately after fabrication and following 3 and 21 days in culture compared to the dimensions of human cervical disc as described by Busscher et al. (2010) and Weiler et al. (2012).

**Time point**	**Height (mm)**	**Diameter (mm)**
Time 0	4.0 ± 0.1	14.0 ± 0.1
Day 3	3.8 ± 0.1	14.0 ± 0.1
Day 21	3.1 ± 0.1	11.2 ± 0.1
Human cervical disc	4.4 ± 1.0	18.0 ± 1.0

*Measurements were taken using a metric ruler to determine the height and diameter of the engineered annulus fibrosus. Data presented as mean ± standard deviation (n = 5)*.

#### Cell Viability and Proliferation

**Figure 7:**
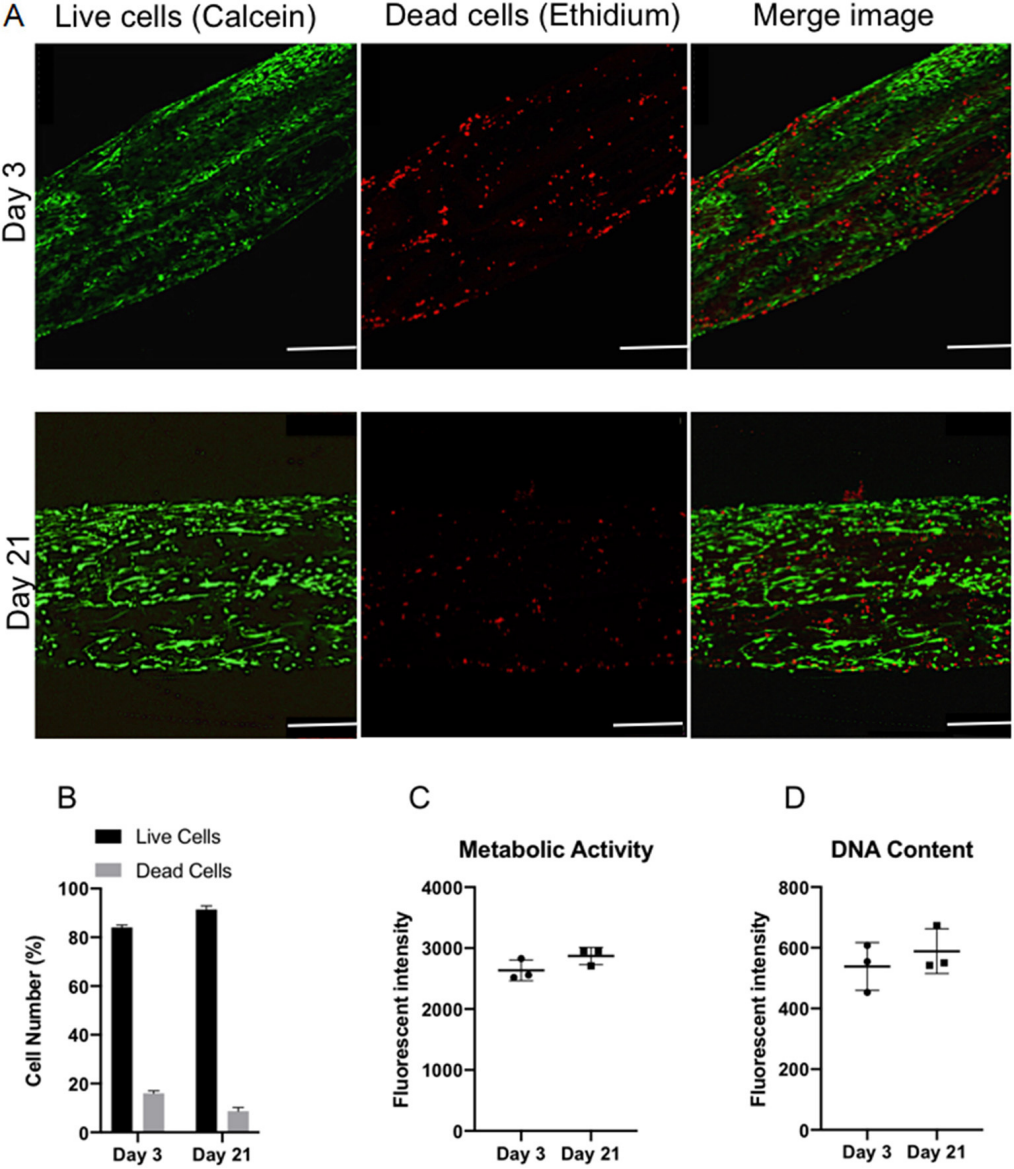
*In vitro* viability of bovine annulus fibrosus (AF) cells within the 3D ringed electrospun PCL:PLLA scaffold after 3 and 21 days. **(A)** Representative confocal images of live cells (green; calcein staining) and dead cells (red; ethidium staining) (*n* = 3; Magnification = x120; Scale bar = 250 μm); **(B)** Semi-quantitative analysis of cell counts taken from three representative Live/Dead images **(C)** Quantitative analysis of metabolic activity (*n* = 3), presented as fluorescence intensity and **(D)** Quantitative analysis of DNA content (*n* = 3), presented as fluorescence intensity. Data reported as mean ± SD with unpaired *t*-test followed by Welch's correction (*p* < 0.05; *n* = 3).

Confocal microscopy was used to image live/dead cells within the ringed lamellar construct after 3 and 21 days in culture (Figure 7A). Representative images exhibited a slight increase in the proportion of live cells (green) (*p* > 0.05) and a decrease in dead cells (red) over time (*p* > 0.05). Importantly, the number of live cells was evidently greater than the number of dead cells for each time point, with ~80% viability at day 3, which increased to ~90% by day 21 (Figure 7B). This slight increase in viability was mirrored with a measurable, yet insignificant, increase in fluorescence intensity after 21 days for cell metabolic activity (8.9%) (Figure 7C) and DNA content (17.6%) (Figure 7D).

#### Cell Distribution and Orientation

**Figure 8:**
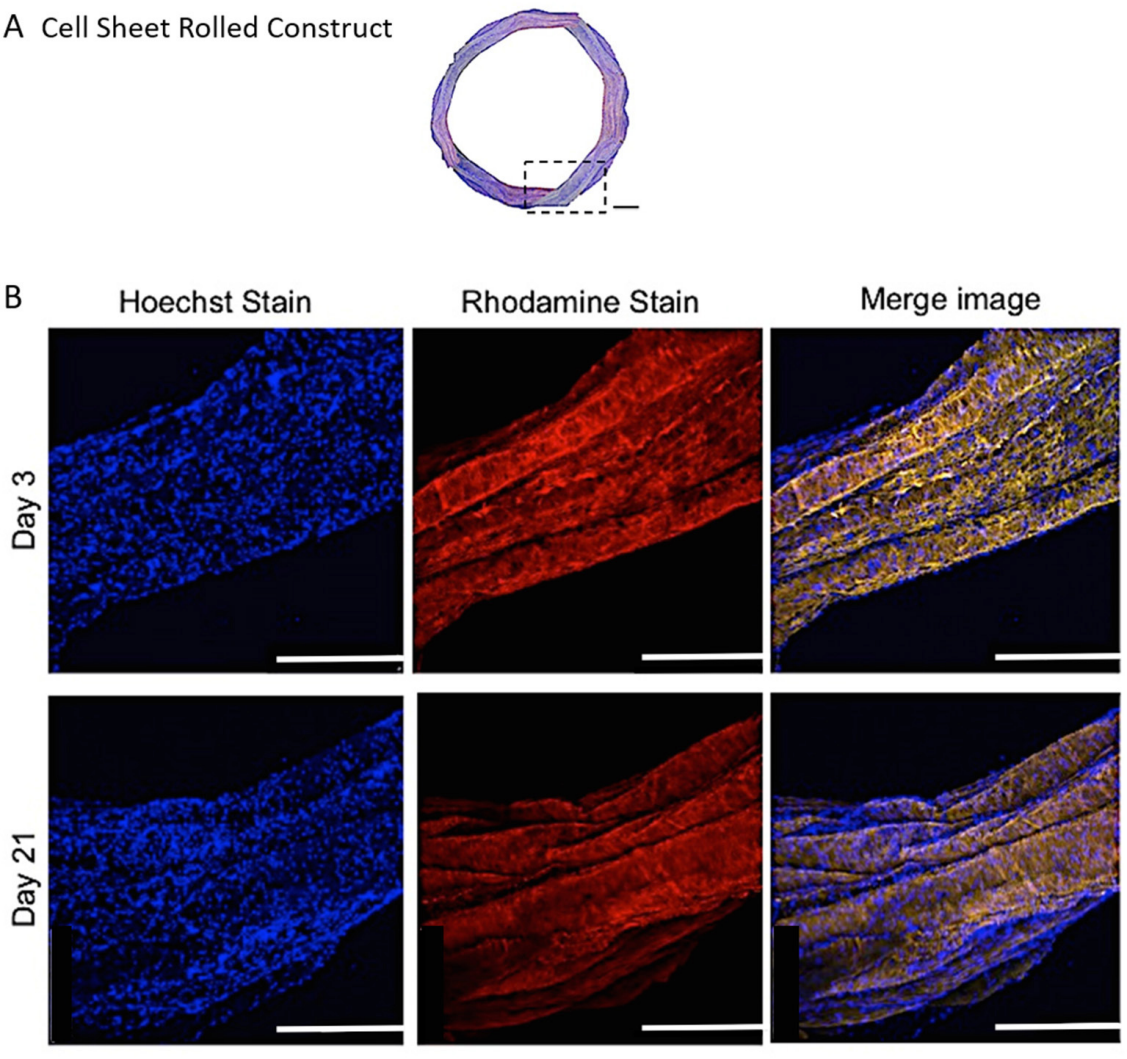
**(A)** A modified (number of images stitched together) confocal microscopy image representing the top view of the PCL:PLLA electrospun 3D ringed lamellar construct (Magnification x25; Scale bar = 1 mm). The dashed square indicates the position used for higher magnification imaging **(B)** Representative confocal microscopy Z-stack projected images after 3 and 21 days for annulus fibrosus (AF) cells: Hoechst staining for cell nuclei (blue), Rhodamine staining for electrospun fibers and merged image (Magnification x60; Scale bar = 500 μm).

Representative confocal images demonstrate the distribution of bovine AF cells within the electrospun ringed lamellar construct after 3 and 21 days of culture (Figure 8). AF cells (nuclei stained blue) were homogeneously distributed within these tubular lamellar constructs and fully surrounded the electrospun fibers, which had been fluorescently labeled with rhodamine (red). By day 21, an increase in the number of cells present on the outer surface of the constructs was visible.

**Figure 9:**
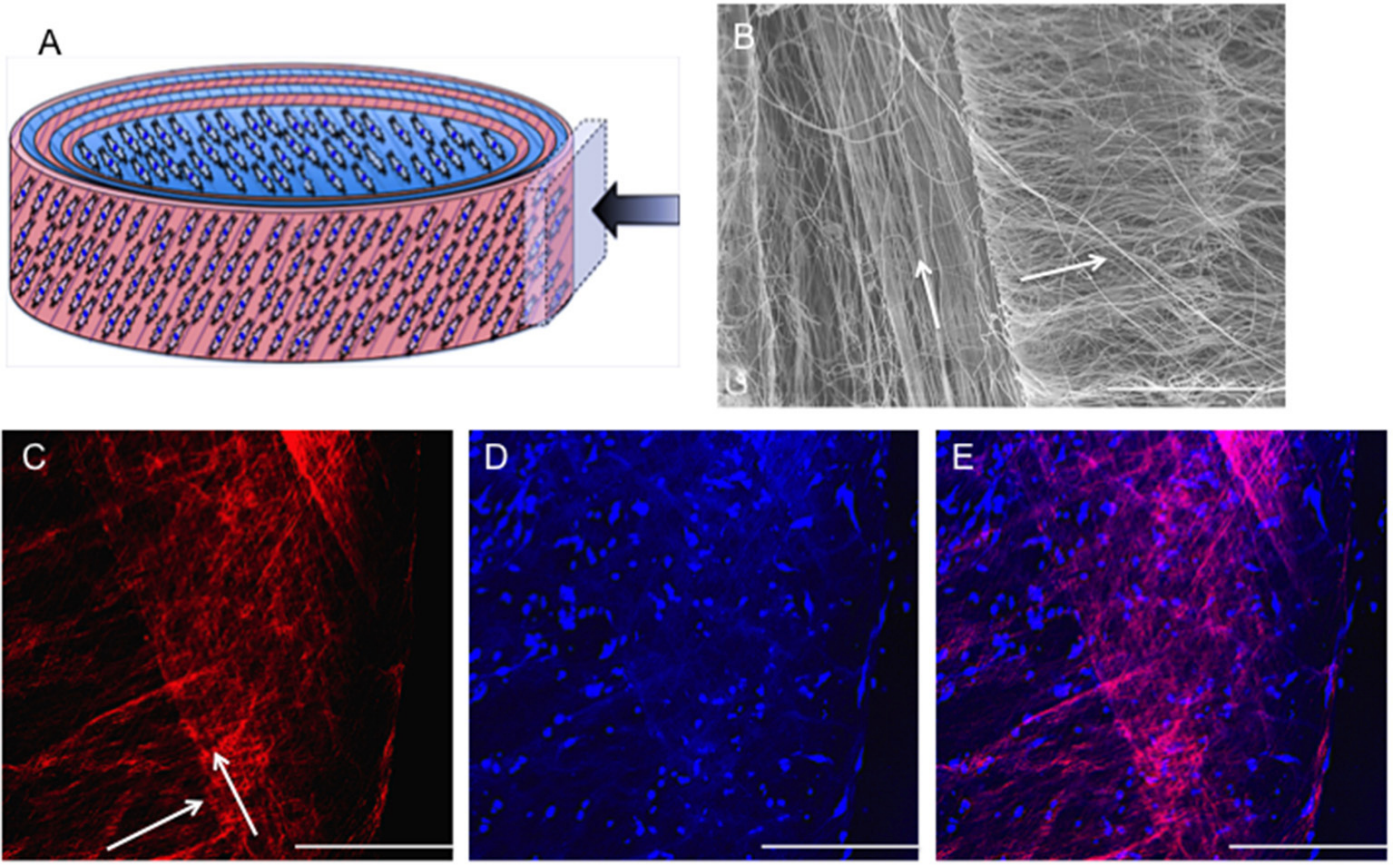
**(A)** Schematic diagram of the cellular 3D ringed lamellar construct and demonstration of the area selected for imaging. **(B)** SEM image demonstrating fiber alignment in opposing directions between adjacent layers (magnification = x500; Scale bar = 100 μm). **(C)** Representative confocal microscopy Z-stack projected image of rhodamine-stained (red) electrospun fibers highlighting the criss-cross lamellar scaffold layers, **(D)** bovine annulus fibrosus cell nuclei stained with Hoechst (blue) present on the adjacent electrospun fiber layers, and **(E)** merged image following 3 days post-rolling (White arrows indicate fiber direction) (Confocal images: magnification x100; Scale bar = 250 μm).

**Figure 10:**
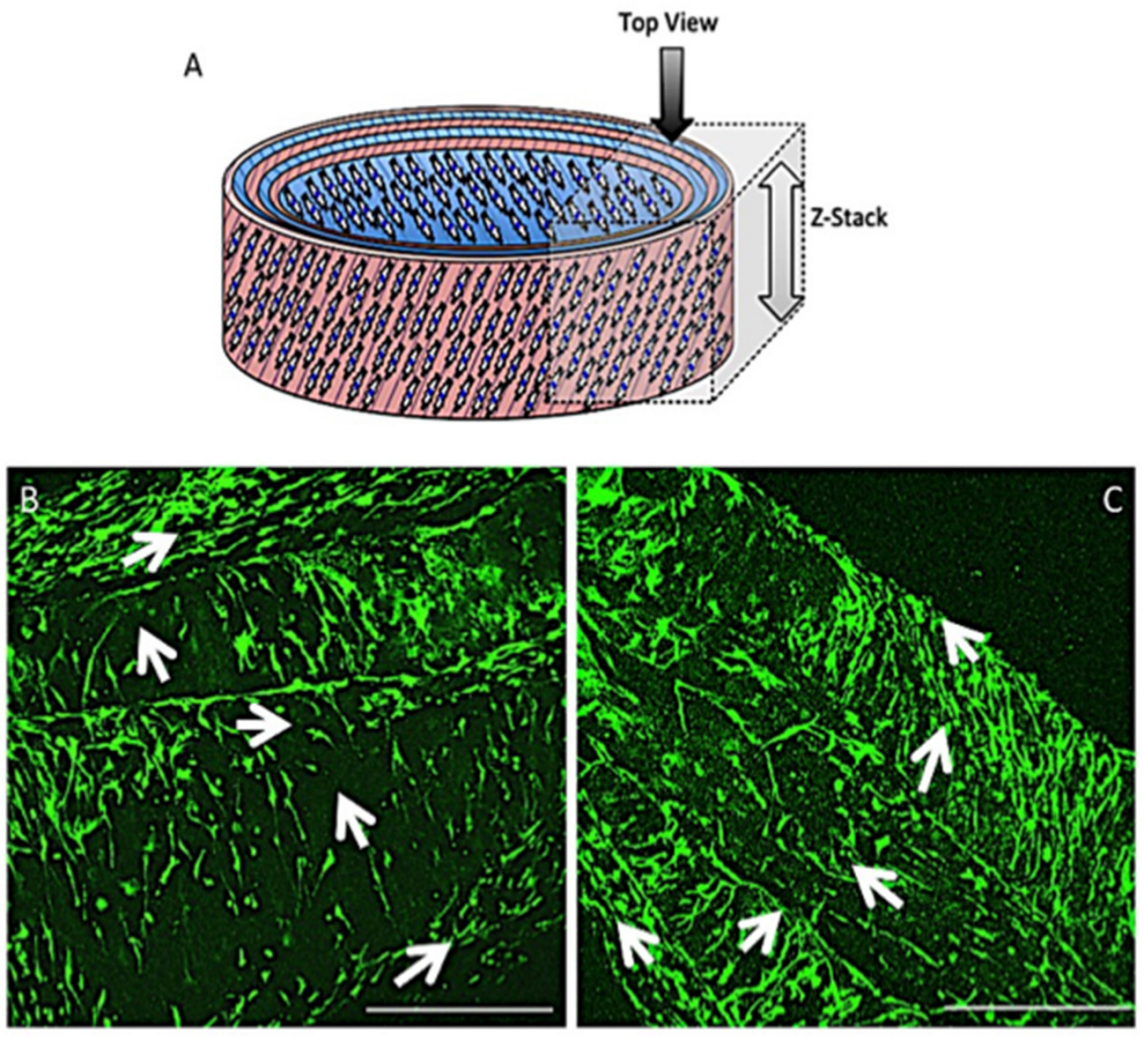
Schematic diagram of the cellular 3D ringed lamellar construct with the direction and area of Z-stack imaging indicated **(A)**. Representative confocal microscopy images for annulus fibrosus (AF) cell orientation within the PCL:PLLA electrospun tubular construct using calcein stain (green) after 3 days **(B)** and 21 days **(C)**. White arrows indicate cells' direction (Scale bars = 500 μm at x65 magnification).

Fiber alignment in opposing directions within adjacent layers of the ringed lamellar construct (Figure 9A) was evident after 3 days in culture using both SEM (Figure 9B) and confocal imaging (Figure 9C). The blue staining for cell nuclei (Figure 9D) showed the AF cells were homogeneously distributed across the aligned rhodaminestained fibers; however, their alignment to the underlying fibers did not clearly present a crisscross configuration (Figure 9E). To directly observe the orientation of AF cells within the rolled construct calcein stain on live cells was performed after 3 and 21 days, which demonstrated their parallel alignment to the underlying fibers and created a cellular crisscross configuration that was maintained over the period of culture (Figure 10).

#### Construct Integrity and ECM Deposition

**Figure 11:**
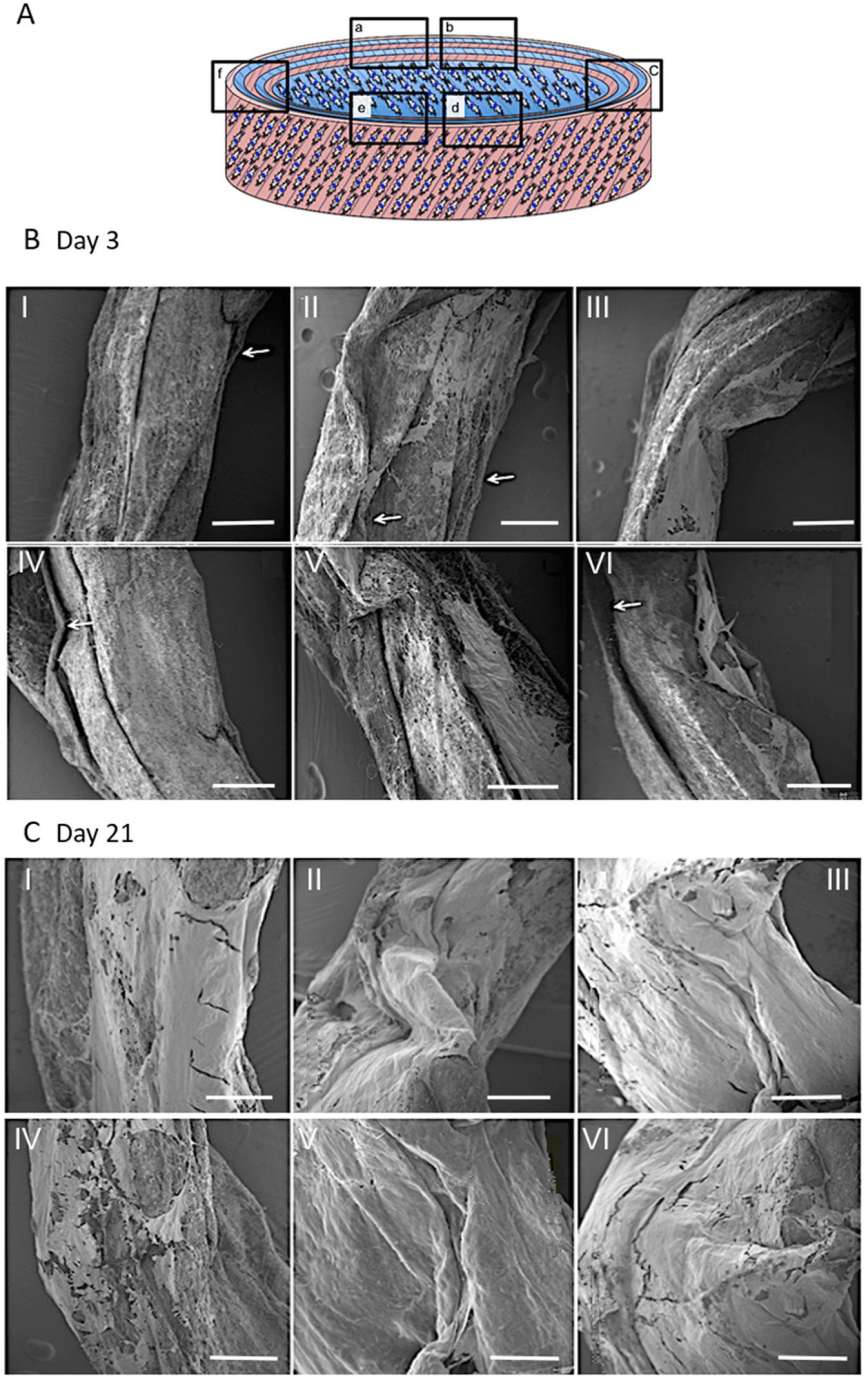
Schematic diagram of the cellular 3D ringed lamellar construct and demonstration of the areas selected for Scanning Electron Microscopy (SEM) imaging **(A)**. Representative SEM images demonstrating the layered tubular construct and deposition of secreted extracellular matrix formation at day 3 **(B)** and day 21 **(C)**. White arrows indicate slight separation between synthetic lamellar sheets in day 3 samples (Magnification x50, scale bar = 500 μm).

SEM was used to examine the structure and morphology of the ECM present within the constructs' layers (Figure 11). The rolled constructs were formed with adjacent lamellar layers in contact with each other; although slight separation between some of the layers was observed at day 3 (Figure 11B). Bridging of cells and secreted ECM across scaffold layers was also evident in certain regions of the ringed construct (Figures 11BI,II,V). By day 21, no lamellar separation was observed and a greater quantity of ECM was apparent with crossover between the layers, suggesting integration of the synthetic lamellae with cells and ECM had occurred (Figure 11C).

**Figure 12:**
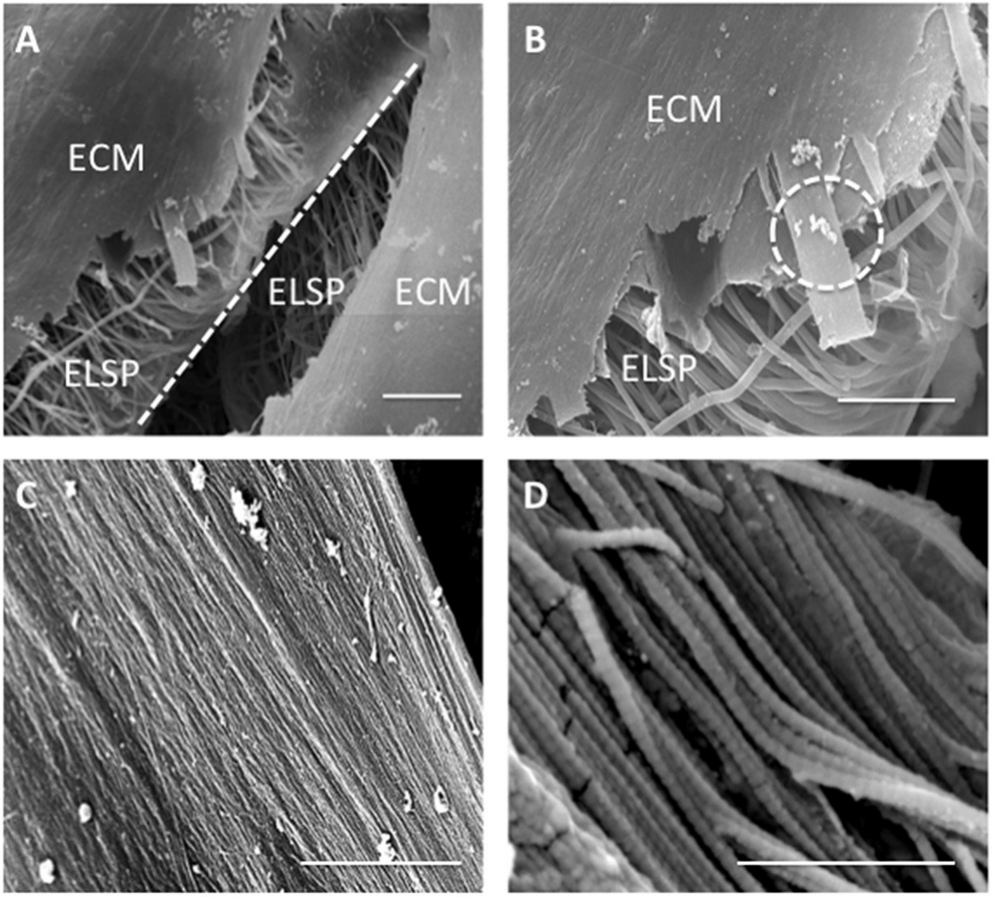
Scanning Electron Microscopy images demonstrating **(A)** extracellular matrix (ECM) formation within the engineered lamellar construct (magnification x1,000, scale bar = 50 μm), **(B)** suspected collagen lamella formation on top of the aligned electrospun fibers scaffold (ELSP) (magnification x4,000, scale bar = 10 μm), **(C)** formation of highly aligned collagen fibers present on the scaffold surface (magnification x10,000, scale bar = 2 μm), and **(D)** collagen fiber morphology demonstrating the clear D-banding characteristic associated with fibrillar collagen) (magnification x120,000, scale bar = 0.3 μm) [White dash-line indicates separation between lamellar construct layers. White dash-circle indicates high magnification image in **(C,D)**].

Higher magnification SEM images demonstrated a thin, sheet-like formation of ECM present at the lamellar interface in between the layers of the construct after 21 days in culture (Figure 12A). This engineered sheet of suspected collagen exhibited a densely packed, organized fibrous structure (Figure 12B). Further study by SEM at x10 k and x120 k magnifications, confirmed this deposited ECM to consist of a closely packed layer of collagen fibers (Figure 12C), which presented a clear D-banding pattern that is characteristic of fibrillar collagen and it's roughened topography (Figure 12D).

#### ECM Immunohistochemistry

**Figure 13:**
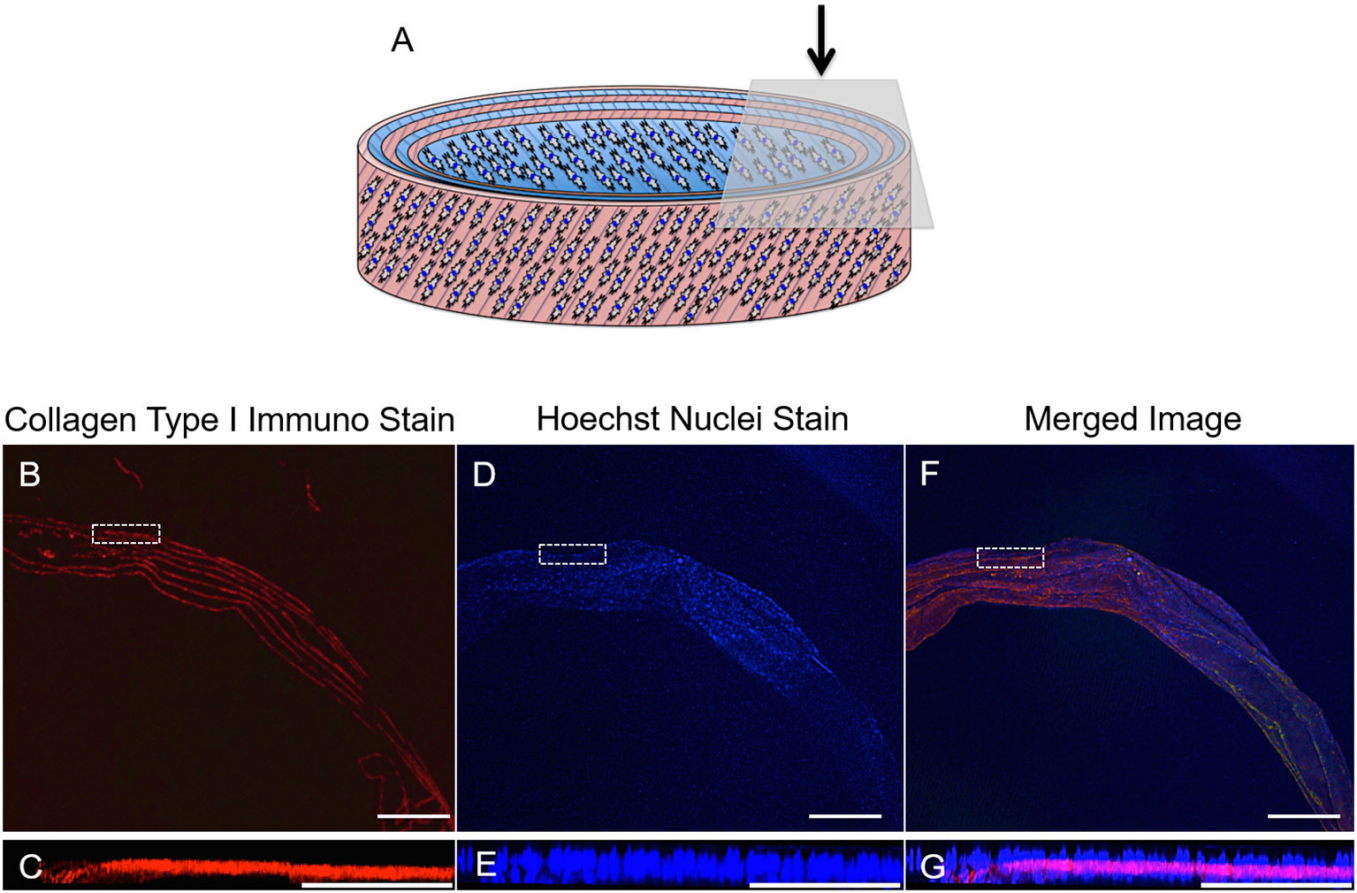
**(A)** Schematic diagram of the cellular 3D ringed lamellar construct and demonstration of the topside region selected for confocal microscopy imaging. **(B,C)** Transverse confocal microscopy images demonstrating positive fluorescent staining of collagen type I (red) present in between the layers of the construct secreted by **(D,E)** annulus fibrosus (AF) cells (nuclei = blue) *in vitro* after 21 days in cell culture media**. (F,G)** Merged image. (Magnification **B,D,F** = x25, Scale bars = 1 mm). The white dashed square indicates the higher magnification imaging (x150) in **(C,E,G)** (Scale bar = 250 μm).

Fluorescence imaging was used to identify the presence of cells and ECM secreted by the AF cells during the 21-day culture period (Figure 13). Positive staining for collagen type I (red) revealed 7 distinct layers overlaying all 6 layers of the rolled synthetic construct. Furthermore, cell nuclei (blue) were apparent on either side of these collagen layers suggesting integration of the synthetic and collagen layers throughout the ringed structure.

## Discussion

### Fabricating Bilayer Scaffolds and *in vitro* Cell Response

Regeneration of the AF through tissue-engineering strategies involves repopulation of the IVD with a cellular scaffold that can direct matrix synthesis to regenerate the disc and treat sufferers of lower back pain. A cellular, bilayer scaffold was successfully obtained by joining two sheets (top and bottom layers) at ±30° fiber direction to generate a superior scaffold mimic of native, outer AF tissue structure. Confocal microscopy and SEM analysis confirmed a criss-cross arrangement of fibers (Figures 3, 4). Seeding of bovine AF cells on individual layers, followed by stacking to create the ±30° bilayer structure and subsequent culture *in vitro* for 14 days demonstrated the unique ability of electrospun fibers to preferentially orient the cells parallel to the main fiber direction. Cell alignment was apparent from day 1 and continued over the duration of study. Furthermore, an observed increase in cell number over time demonstrated biocompatibility of the bilayer. However, imaging of the bottom layer at day 7 revealed cells to be oriented in opposite directions to one another, which would suggest close apposition with the upper scaffold layer and influence of the opposing fiber direction on cell morphology. The continued ability of aligned electrospun fibers to provide contact guidance cues to the cells within the bilayer scaffold, suggests the orientation of ECM secreted by the AF cells will similarly be arranged parallel to the cell, which should then mimic the ECM arrangement *in vivo*. These data are consistent with previous studies, including Nerurkar et al. (2009), Martin et al. (2014), Yang et al. (2011), and Fotticchia et al. (2018), who created multi-layer structures by stacking individual sheets of cell-seeded electrospun fibers.

Culture of AF cells within bilayer scaffolds over the 2-week period also resulted in significant changes in tensile properties (Figure 5). Compared to acellular scaffolds, Young's modulus was significantly increased following the inclusion of cells after 14 days culture (84.7 ± 7.0 MPa, 41% increase), suggesting the network of cells and their secreted ECM have had a positive impact on scaffold stiffness. Interestingly, the stiffness after 14 days resulted in a 61% difference compared to the upper end of human AF lamella, which has previously been shown to range from 18 to 45 MPa (Ebara et al., 1996; Elliott and Setton, 2001). However, the measured mechanical properties of the native tissue depends on age, harvest site, tissue size/thickness (ranging from 2.3 to 5 mm) (Inoue and Takeda, 1975; Marchand and Ahmed, 1990), angles between lamellae (ranging from 30° to 60°) (Hickey and Hukins, 1980; Tsuji et al., 1993) and also the testing configuration.

In terms of ultimate tensile strength, incorporation and culture of AF cells resulted in a positive impact on scaffold strength being 70% stronger after 7 days (8.4 ± 0.6 MPa), and within the range of reported human AF lamella (3.6–10.0 MPa) (Skaggs et al., 1994), which continued following 14 days in culture. This again suggests integration between the bilayer scaffold and the cells and their deposited matrix connecting in-between scaffold fibers and layers; and is stronger than our previously reported tensile data for cell-loaded single layer scaffolds (Shamsah et al., 2019). Moreover, analysis of the maximum strain indicated the presence of cells and ECM contributed to a reduction in scaffold elongation after 14 days in culture, which may be attributed to friction between the matrix and the two layers causing raised shear stresses that need to be overcome whilst undergoing tensile loading (Nerurkar et al., 2009; Horgan and Murphy, 2017). Thus, the presence of cells and matrix may help to resist deformation resulting in an increase in scaffold strength, which is similar behavior to the native AF tissue as described in the literature (Nerurkar et al., 2010, 2011; Driscoll et al., 2011, 2013).

It should be noted that these scaffolds were initially fixed in 10% NBF, which may have an impact on the mechanical properties due to crosslinking. However, several published studies comparing fixed to live cells for multiple cell types suggest this impact to be negligible (Kim, 2008; Grimm et al., 2014; Zhang et al., 2017).

### Fabricating 3D AF Tissue Biomimics Using CSRS and *in vitro* Cell Response

To recapitulate the outer layers of AF tissue from cellular aligned electrospun bilayer scaffolds a CSRS was used for this specific application for the first time. This allowed an automated rolling protocol to be applied to the cellular bilayer scaffold, which resulted in the fabrication of a 3D AF tissue mimic. To aid assembly and maintain the 3D ringed structure post-rolling, fibrin glue was applied at the end of the remaining bilayer scaffold to be rolled, which prevented construct disassembly (Figures 2B,C). These AF tissue constructs were subsequently immersed in culture media and incubated for 3 weeks. During this time, all of the engineered tubular lamellar constructs remained intact with no sign of delamination or structural collapse.

As such, viable multilayer ringed constructs, comprising six layers of cellular bilayers were successfully created. However, one of the challenges when aiming to engineer AF tissue is generating a tubular lamellar construct that is of appropriate human size and geometry. When measured, the fabricated construct in this study was found to possess dimensions close to human cervical disc. The diameter of the engineered AF tissue construct reached 14 ± 0.1 mm, with a 25% difference compared to human cervical disc diameter (18 mm) (Busscher et al., 2010; Weiler et al., 2012). In order to achieve closer approximation to full sized human cervical IVDs, the CSRS can be easily adjusted to accommodate different mandrel diameters and longer lengths of the initial bilayer scaffold could also be fabricated, which would allow larger dimensions to be achieved. This has been previously demonstrated by Othman et al. (2015), where tubular structures were fabricated using the CSRS with different sized mandrels to create a range of scaffold dimensions, which were comparable to the native tissue of interest, such as the aorta and trachea. Moreover, contraction of the cellular lamellar construct over time also needs to be considered and a larger mandrel may need to be used during the rolling process to allow for this shrinkage. This is based on the change in dimensions of the ringed construct after 21 days in culture, where construct height reduced by 18.4% and diameter by 20.0% when compared to construct dimensions at 3 days. These changes in construct dimensions during culture are likely to be due to a greater number of cells contracting on the material and on their secreted ECM (Knight and Przyborski, 2015; Goyal et al., 2017). Furthermore, these ringed constructs lacked a gelatinous NP core, which may also have helped to stabilize the structure.

Biological investigation provided a direct indication of AF cell viability within the 3D lamellar construct. Over the culture period, cells demonstrated good viability with 91.3 ± 1.5% of live cells counted at 21 days, and coupled with slight, though insignificant, increases in cell metabolic activity and DNA content, demonstrates that this large, 3D scaffold did not impede cellular function and supported nutrient exchange through the electrospun fiber layers (Figure 7). Confocal microscopy images further revealed the AF cells to be well-spread and homogeneously covered the entire surface of the construct after 21 days, as indicated by cell nuclei distribution (Figure 8). In addition, the AF cells seeded on aligned electrospun bilayers scaffold and then rolled to create 3D ringed structures demonstrated specific crisscross orientations, which mimic AF cell arrangement *in vivo*. This demonstrates the continued ability of electrospun aligned fibers to provide contact guidance cues to the attached cells despite cells being cultured vertically within the tubular structure, and is in agreement with studies by Koepsell et al. (2011) and Wismer et al. (2014).

Over the 3-week culture period studied, ECM synthesis was evident from SEM images taken at a range of magnifications (Figure 12). Matrix deposition was apparent at day 3 but a greater quantity was observed after 21 days. SEM images indicated areas of delamination for several of the constructs at day 3, but these had disappeared after 21 days, which is likely due to the cells increasing in number and also greater secretion of ECM over time, therefore allowing scaffold layers to form integrated, bridge-like connections. In addition, SEM imaging between scaffold layers revealed lamellar-like depositions at the electrospun layer interfaces, suggesting the seeded cells—guided by the electrospun fibers—secreted similarly organized ECM (Figure 11). High magnification SEM images indicated the ECM deposition resulted in highly aligned, collagen-rich lamellae with a clear D-banding pattern, which is characteristic of collagen type I fibers (Figure 12). This is further supported by immunofluorescence staining, which revealed this secreted matrix to be collagen type I (Figure 13). These findings indicate this fabricated cell-containing, ringed lamellar construct provides a 3D biomimetic architecture that is appropriate to support formation of AF tissue.

Overall, this study demonstrates the ability to replicate the structural hierarchy of the outer AF using bilayer scaffolds, formed by stacking together two single-layer scaffolds of PCL:PLLA blended electrospun sheets with opposing fiber directions that had been previously seeded with bovine AF cells. Following 2 weeks in culture, these cells demonstrated a specific crisscross orientation, which closely mimics AF cell arrangement *in vivo*. Changes in mechanical behavior were observed for these cellular bilayers compared to acellular constructs, where dual fiber sheets with cells and deposited matrix, yielded significant increases in tensile modulus and strength at break. This provides further evidence that a cellular bilayer scaffold with layers of alternating angle-ply fiber orientation is an important factor when considering the final mechanical properties of a tissue engineered scaffold. Incorporating the CSRS has enabled the transition of essentially 2D electrospun bilayer scaffolds into 3D ringed, lamellar constructs and is the first application of this technology for AF tissue mimicry. These 3D constructs supported viable AF cells after 3 weeks in *in vitro* culture and secretion of oriented ECM lamellae that were positive for collagen type I. These data therefore demonstrate the potential of the CSRS as a promising technology for the development of tissue engineered AF and, ultimately, clinically relevant IVD structures. The development of a whole IVD could potentially be achieved through the combination of a hydrogel core, such as self-assembling peptides (Wan et al., 2016) to provide a mimic of the nucleus pulposus, which may then also integrate with the inner layers of this 3D-ringed electrospun structure, with multiple layers providing a distinct transition from inner to outer AF zones.

## Conclusion

This is the first study using an automated cell sheet rolling system to create innovative, 3D ringed electrospun structures, where fibers are held in an angle-ply configuration to mimic the annulus fibrosus. These scaffolds support cell viability and orientation, desirable tensile properties and secretion of collagen type I, which is the principal component of AF tissue.

This research demonstrates the first step toward creating clinically-relevant scaffolds for tissue engineering applications, such as whole intervertebral disc replacement. Further studies will focus on the application of dynamic loading to better mimic *in vivo* conditions and cell response in terms of matrix composition and deposition, and impact on mechanical properties in order to determine the structure's utility as a functional, tissue engineered device.

## Data Availability Statement

The datasets generated for this study are available on request to the corresponding author.

## Author Contributions

The first draft of the manuscript was written by AS. All authors contributed to the conception and design of the study, manuscript revision, and read and approved the submitted version.

### Conflict of Interest

The authors declare that the research was conducted in the absence of any commercial or financial relationships that could be construed as a potential conflict of interest.

## References

[B1] Abrámoff M. D. Magalhães P. J. Ram S. J. (2004). Image processing with ImageJ. Biophoton. Int. 11, 36–42. 10.1201/9781420005615.ax4

[B2] Bosworth L. A. Downes S. (2010). Physicochemical characterisation of degrading polycaprolactone scaffolds. Polym. Degrad. Stab. 95, 2269–2276. 10.1016/j.polymdegradstab.2010.09.007

[B3] Buckley C. T. Hoyland J. A. Fujii K. Pandit A. Iatridis J. C. Grad S. (2018). Critical aspects and challenges for intervertebral disc repair and regeneration — harnessing advances in tissue engineering. Spine 1:e1029. 10.1002/jsp2.1029 30895276 PMC6400108

[B4] Busscher I. Ploegmakers J. J. Verkerke G. J. Veldhuizen A. G. (2010). Comparative anatomical dimensions of the complete human and porcine spine. Eur. Spine J. 19, 1104–1114. 10.1007/s00586-010-1326-9 20186441 PMC2900026

[B5] Chen L. Bai Y. Liao G. Peng E. Wu B. Wang Y. (2013). Electrospun poly(L-Lactide)/poly(ε-Caprolactone) blend nanofibrous scaffold: characterization and biocompatibility with human adipose-derived stem cells. PLoS ONE 8, 14–16. 10.1371/journal.pone.0071265 23990941 PMC3753307

[B6] Chu G. Shi C. Wang H. Zhang W. Yang H. Li B. (2018). Strategies for annulus fibrosus regeneration: from biological therapies to tissue engineering. Front. Bioeng. Biotechnol. 6, 1–13. 10.3389/fbioe.2018.00090 30042942 PMC6048238

[B7] Driscoll T. P. Nakasone R. H. Szczesny S. E. Elliott D. M. Mauck R. L. (2013). Biaxial mechanics and inter-lamellar shearing of stem-cell seeded electrospun angle-ply laminates for annulus fibrosus tissue engineering. J. Orthopaed. Res. 31, 864–70. 10.1002/jor.22312 23335319

[B8] Driscoll T. P. Nerurkar N. L. Jacobs N. T. Elliott D. M. Mauck R. L. (2011). Fiber angle and aspect ratio influence the shear mechanics of oriented electrospun nanofibrous scaffolds. J. Mech. Behav. Biomed. Mater. 4, 1627–1636. 10.1016/j.jmbbm.2011.03.022 22098865 PMC3221959

[B9] Ebara S. Iatridis J. C. Setton L. A. Foster R. J. Mow V. C. Weidenbaum M. (1996). Tensile properties of nondegenerate human lumbar anulus fibrosus. Spine 21, 452–461. 10.1097/00007632-199602150-00009 8658249

[B10] Elliott D. M. Setton L. A. (2001). Anisotropic and inhomogeneous tensile behavior of the human anulus fibrosus: experimental measurement and material model predictions. J. Biomech. Eng. 123, 256–263. 10.1115/1.1374202 11476369

[B11] Fotticchia A. Demirci E. Lenardi C. Liu Y. (2018). Cellular response to cyclic compression of tissue engineered intervertebral disk constructs composed of electrospun polycaprolactone. J. Biomech. Eng. 140:061002 10.1115/1.4039307 29450477

[B12] Goyal R. Guvendiren M. Freeman O. Mao Y. Kohn J. (2017). Optimization of polymer-ECM composite scaffolds for tissue engineering: effect of cells and culture conditions on polymeric nanofiber mats. J. Funct. Biomater. 8:1. 10.3390/jfb8010001 28085047 PMC5371874

[B13] Grimm K. B. Oberleithner H. Fels J. (2014). Fixed endothelial cells exhibit physiologically relevant nanomechanics of the cortical actin web. Nanotechnology. 25:215101. 10.1088/0957-4484/25/21/215101. 24786855

[B14] Hickey D. S. Hukins D. W. (1980). X-ray diffraction studies of the arrangement of collagenous fibres in human fetal intervertebral disc. J. Anat. 131, 81–90. 7440405 PMC1233288

[B15] Holzapfel G. A. Schulze-Bauer C. A. J. Feigl G. Regitnig P. (2005). Single lamellar mechanics of the human lumbar anulus fibrosus. Biomech. Model. Mechanobiol. 3, 125–140. 10.1007/s10237-004-0053-8 15778871

[B16] Horgan C. O. Murphy J. G. (2017). Fiber orientation effects in simple shearing of fibrous soft tissues. J. Biomech. 64, 131–135. 10.1016/j.jbiomech.2017.09.018 29033002

[B17] Inoue H. Takeda T. (1975). Three-dimensional observation of collagen framework of lumbar intervertebral discs. Acta Orthopaed. Scand. 46, 949–956. 10.3109/17453677508989283 1211132

[B18] Kandel R. Santerre P. Massicotte E. Hurtig M. (2014). Tissue engineering of the intervertebral disc, in The intervertebral disc: molecular and structural studies of the disc in health and disease, eds Shapiro I. Risbud M. (Wien: Springer), 417–433.

[B19] Kim G. H. (2008). Electrospun PCL nanofibers with anisotropic mechanical properties as a biomedical scaffold. Biomed. Mater. 3:025010. 10.1088/1748-6041/3/2/025010. 18458365

[B20] Knight E. Przyborski S. (2015). Advances in 3D cell culture technologies enabling tissue-like structures to be created *in vitro*. J. Anat. 227, 746–756. 10.1111/joa.12257 25411113 PMC4694114

[B21] Koepsell L. Zhang L. Neufeld D. Fong H. Deng Y. (2011). Electrospun nanofibrous polycaprolactone scaffolds for tissue engineering of annulus fibrosus. Macromol. Biosci. 11, 391–399. 10.1002/mabi.201000352 21080441

[B22] Long R. G. Torre O. M. Hom W. W. Assael D. J. Iatridis J. C. (2016). Design requirements for annulus fibrosus repair: review of forces, displacements, and material properties of the intervertebral disk and a summary of candidate hydrogels for repair. J. Biomech. Eng. 138:021007. 10.1115/1.4032353 26720265 PMC4844119

[B23] Marchand F. Ahmed A. M. (1990). Investigation of the laminate structure of lumbar disc anulus fibrosus. Spine 15, 402–410. 10.1097/00007632-199005000-00011 2363068

[B24] Martin J. T. Milby A. H. Chiaro J. A. Kim D. H. Hebela N. M. Smith L. J.. (2014). Translation of an engineered nanofibrous disc-like angle-ply structure for intervertebral disc replacement in a small animal model. Acta Biomater. 10, 2473–2481. 10.1016/j.actbio.2014.02.024 24560621 PMC4412172

[B25] Nerurkar N. L. Baker B. M. Sen S. Wible E. E. Elliott D. M. Mauck R. L. (2009). Nanofibrous biologic laminates replicate the form and function of the annulus fibrosus. Nat. Mat. 8, 986–992. 10.1038/nmat2558 19855383 PMC3415301

[B26] Nerurkar N. L. Mauck R. L. Elliott D. M. (2011). Modeling interlamellar interactions in angle-ply biologic laminates for annulus fibrosus tissue engineering. Biomech. Model. Mechanobiol. 10, 973–984. 10.1007/s10237-011-0288-0 21287395 PMC3513349

[B27] Nerurkar N. L. Sen S. Huang A. H. Elliott D. M. Mauck R. L. (2010). Engineered disc-like angle-ply structures for intervertebral disc replacement. Spine 35, 867–873. 10.1097/BRS.0b013e3181d74414 20354467 PMC3421837

[B28] Othman R. Morris G. E. Shah D. A Hall S. Hall G. Wells K.. (2015). An automated fabrication strategy to create patterned tubular architectures at cell and tissue scales. Biofabrication 7:025003. 10.1088/1758-5090/7/2/025003 25869447

[B29] Raj P. P. (2008). Intervertebral disc: anatomy-physiology-pathophysiology-treatment. Pain Pract. 8, 18–44. 10.1111/j.1533-2500.2007.00171.x 18211591

[B30] Schutgens E. M. Tryfonidou M. A. Smit T. H. Öner F. C. Krouwels A. Ito K.. (2015). Biomaterials for intervertebral disc regeneration: past performance and possible future strategies. Eur. Cells Mater. 30, 210–231. 10.22203/eCM.v030a15 27227695

[B31] Shamsah A. H. Cartmell S. H. Richardson S. M. Bosworth L. A. (2019). Mimicking the annulus fibrosus using electrospun polyester blended scaffolds. Nanomaterials 9:E537. 10.3390/nano9040537 30987168 PMC6523918

[B32] Skaggs D. L. Weidenbaum M. Iatridis J. C. Ratcliffe A. Mow V. C. (1994). Regional variation in tensile properties and biochemical composition of the human lumbar anulus fibrosus. Spine 19, 1310–1319. 10.1097/00007632-199406000-00002 8066509

[B33] Tsuji H. Hirano N. Ohshima H. Ishihara H. Terahata N. Motoe T. (1993). Structural variation of the anterior and posterior anulus fibrosus in the development of human lumbar intervertebral disc. Spine 18, 204–210. 10.1097/00007632-199302000-00006 8441935

[B34] van Uden S. Silva-Correia J. Oliveira J. M. Reis R. L. (2017). Current strategies for treatment of intervertebral disc degeneration: substitution and regeneration possibilities. Biomater. Res. 21, 1–19. 10.1186/s40824-017-0106-6 29085662 PMC5651638

[B35] Wan S. Borland S. Richardson S. M. Merry C. L. R. Saiani A. Gough J. E. (2016). Self-assembling peptide hydrogel for intervertebral disc tissue engineering. Acta Biomater. 46, 29–40. 10.1016/j.actbio.2016.09.033 27677593

[B36] Weiler C. Schietzsch M. Kirchner T. Nerlich A. G. Boos N. Wuertz K. (2012). Age-related changes in human cervical, thoracal and lumbar intervertebral disc exhibit a strong intra-individual correlation. Eur. Spine J. 21 (Suppl. 6), 810–818. 10.1007/s00586-011-1922-3 21837413 PMC3535216

[B37] Wismer N. Grad S. Fortunato G. Ferguson S. J. Alini M. Eglin D. (2014). Biodegradable electrospun scaffolds for annulus fibrosus tissue engineering: effect of scaffold structure and composition on annulus fibrosus cells *in vitro*. Tissue Eng. Part A 20, 672–682. 10.1089/ten.tea.2012.0679 24131280

[B38] Yang Y. Wimpenny I. Ahearne M. (2011). Portable nanofiber meshes dictate cell orientation throughout three-dimensional hydrogels. Nanomedicine 7, 131–136. 10.1016/j.nano.2010.12.011 21272664

[B39] Zhang C. Shi J. Wang W. Xi N. Wang Y. Liu L. (2017). Simultaneous measurement of multiple mechanical properties of single cells using AFM by indentation and vibration. IEEE Trans. Biomed. Eng. 64:2771–2780. 10.1109/TBME.2017.2674663. 28252389

